# Optimizing theranostics chatbots with context-augmented large language models

**DOI:** 10.7150/thno.107757

**Published:** 2025-04-21

**Authors:** Pia Koller, Christoph Clement, Albert van Eijk, Robert Seifert, Jingjing Zhang, George Prenosil, Mike M. Sathekge, Ken Herrmann, Richard Baum, Wolfgang A. Weber, Axel Rominger, Kuangyu Shi

**Affiliations:** 1 Informatics, Ludwig-Maximilians-University, Geschwister-Scholl-Platz 1, Munich, 80539, Germany.; 2 ITM Radiopharma, Walther-Von-Dyck Str. 4, Garching, 85748, Bavaria, Germany.; 3 Department of Nuclear Medicine, Bern University Hospital, University of Bern, Freiburgstrasse 20, Bern, 3010, Switzerland.; 4 Yong Loo Lin School of Medicine, National University of Singapore, 10 Medical Dr, Singapore, 117597, Singapore.; 5 Nuclear Medicine, University of Pretoria, Private Bag x 20, 0028, Hatfield, South Africa.; 6 Department of Nuclear Medicine, University of Duisburg-Essen, and German Cancer Consortium (DKTK)-University Hospital Essen, Essen, Germany.; 7 National Center for Tumor Diseases (NCT), NCT West, Germany.; 8 International Centers for Precision Oncology (ICPO), CURANOSTICUM Wiesbaden-Frankfurt at DKD Helios Klinik, Aukammallee 33, Wiesbaden, 65191, Germany.; 9 Department of Nuclear Medicine, TUM University Hospital, Technical University Munich, Bavarian Cancer Research Center, Ismaningerstr. 22, Munich, 81675, Germany.; 10 Chair for Computer-aided Medical Procedure, School of Computation, Information & Technology, Technical University Munich, Boltzmannstr. 3, Garching, 85748, Germany.; The corresponding author is the first author of this journal paper, which is based on her research conducted during her master's thesis. She is currently a master's student in Computer Science at Ludwig-Maximilians-University in Munich.

**Keywords:** Large Language Models, Contextual Augmentation, Retrieval Augmented Generation, Nuclear Medicine, Theranostics

## Abstract

**Introduction**: Nuclear medicine theranostics is rapidly emerging, as an interdisciplinary therapy option with multi-dimensional considerations. Healthcare Professionals do not have the time to do in depth research on every therapy option. Personalized Chatbots might help to educate them. Chatbots using Large Language Models (LLMs), such as ChatGPT, are gaining interest addressing these challenges. However, chatbot performances often fall short in specific domains, which is critical in healthcare applications.

**Methods**: This study develops a framework to examine the use of contextual augmentation to improve the performance of medical theranostic chatbots to create the first theranostic chatbot. Contextual augmentation involves providing additional relevant information to LLMs to improve their responses. We evaluate five state-of-the-art LLMs on questions translated into English and German. We compare answers generated with and without contextual augmentation, where the LLMs access pre-selected research papers via Retrieval Augmented Generation (RAG). We are using two RAG techniques: Naïve RAG and Advanced RAG.

**Results**: A user study and LLM-based evaluation assess answer quality across different metrics. Results show that Advanced RAG techniques considerably enhance LLM performance. Among the models, the best-performing variants are CLAUDE 3 OPUS and GPT-4O. These models consistently achieve the highest scores, indicating robust integration and utilization of contextual information. The most notable improvements between Naive RAG and Advanced RAG are observed in the GEMINI 1.5 and COMMAND R+ variants.

**Conclusion**: This study demonstrates that contextual augmentation addresses the complexities inherent in theranostics. Despite promising results, key limitations include the biased selection of questions focusing primarily on PRRT, the need for comprehensive context documents. Future research should include a broader range of theranostics questions, explore additional RAG methods and aim to compare human and LLM evaluations more directly to enhance LLM performance further.

## Introduction

Nuclear medicine has experienced groundbreaking advancements over the past decade. The success of targeted radiopharmaceutical therapy is transforming nuclear medicine from a primarily diagnostic field to an integral component of oncological treatment 2. This evolution involves multi-dimensional considerations, encompassing clinical, physical, pharmaceutical, biological, and environmental perspectives 19. The rapid development of new radioisotopes, molecules, and combined treatment regimens presents significant challenges for professionals, patients, the public, and regulatory agencies 17.

Simultaneously, the emergence of Large Language Models (LLMs) has revolutionized many fields, offering powerful tools to integrate multi-dimensional information sources and enhance complex knowledge translation. Chatbots that use LLMs provide access to healthcare information, assist with appointment scheduling, and offer preliminary medical advice, thus improving the efficiency of healthcare systems and providing patients with timely support 1. A great benefit LLMs provide for healthcare professionals is for example personalized education, with personalized answers to specific questions 6. However, LLMs face the challenge of hallucination and the potential for misinformation if not properly integrated with accurate knowledge 20, posing risks for clinical practice, radiopharmaceutical development, and radiation protection. One major limitation is their limited capability for handling complex medical issues 14,16. While AI-powered chatbots are commonly used for patient interactions, there is a gap for chatbots specifically designed as educational tools for healthcare professionals (HCPs) in theranostics and nuclear medicine. HCPs often lack sufficient time to conduct in-depth research on emerging topics of nuclear medicine. An educational chatbot can help HCPs to quickly understand certain procedures or surgeries. These chatbots will not replace literature searches but rather provide an overview of a topic.

This study focuses on enhancing the performance of LLM-powered medical chatbots in the specialized field of theranostics and nuclear medicine by utilizing Retrieval Augmented Generation (RAG) techniques, which were first introduced by Lewis et al. (2021). This is achieved by comparing responses generated by LLMs with and without contextual augmentation and assessing their quality through both human and LLM evaluations. For the LLM responses with contextual augmentation, we are using two RAG techniques, Naïve RAGs and Advanced RAGs. We aim to evaluate how assisting LLMs with a set of research papers influences the quality and accuracy of chatbot responses.

The contribution of this research involves the design, implementation and evaluation of a framework to evaluate LLM performance by humans and other LLMs as a judge. It offers a robust approach for assessing and comparing the performance of various LLMs in medical contexts. The results demonstrate how contextual augmentation through RAG techniques can improve the accuracy and relevance of chatbot responses.

## Methods

### Contextual Augmentation with Retrieval Augmented Generation

A technique like RAG allows LLMs to access and incorporate information about the latest research and clinical guidelines without any additional training 15. We chose a set of 197 pre-selected research papers 11 on theranostics and nuclear medicine through a pragmatic, though not strictly systematic, approach to improve the accuracy and relevance of LLM responses 10.

**Figure 1:**
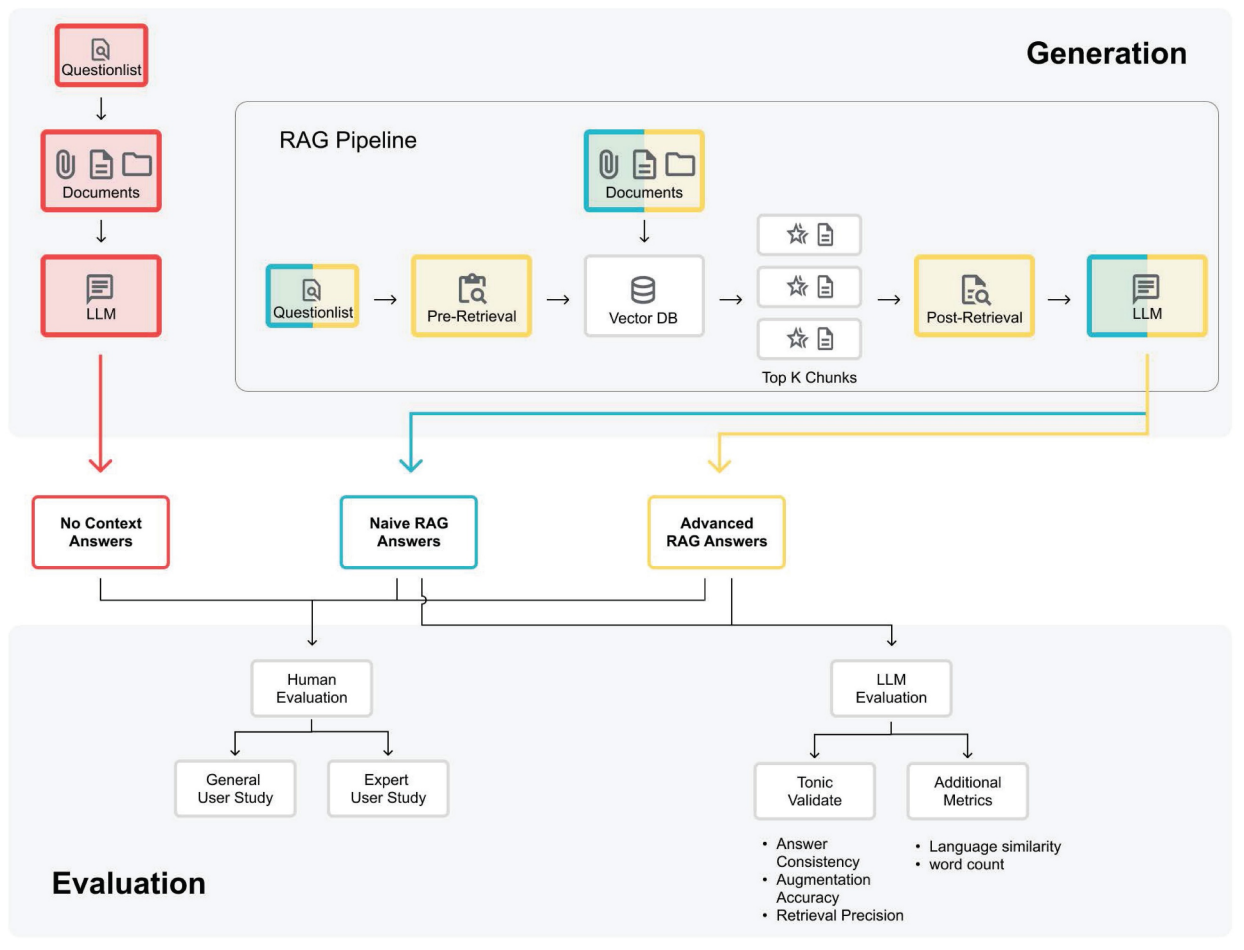
Pipeline for Generation and Evaluation of Theranostic Chatbot Answers: A three-track system illustrates the generation and evaluation of No Context Answers (red), Naïve RAG Answers (blue), and Advanced RAG Answers (yellow). The colored components represent interchangeable parts, enabling the evaluation of new models, questions, context lists, pre- and post-retrieval methods, which ensures adaptability (The RAG Pipeline part adapted from Bainiaksina (2024) 3).

RAG systems operate through a process that includes indexing, retrieval, and generation. The indexing phase involves converting raw data into a uniform format, segmenting it into smaller chunks, and encoding these chunks into a vector database (see RAG Pipeline in **Figure 1**). During retrieval, the user query is transformed into a vector representation, and similarity scores are computed to prioritize the top k chunks most relevant to the query. In the generation phase, the model synthesizes the query and the selected documents into a single prompt, generating a response based on the retrieved context 9,15.

**Figure 2:**
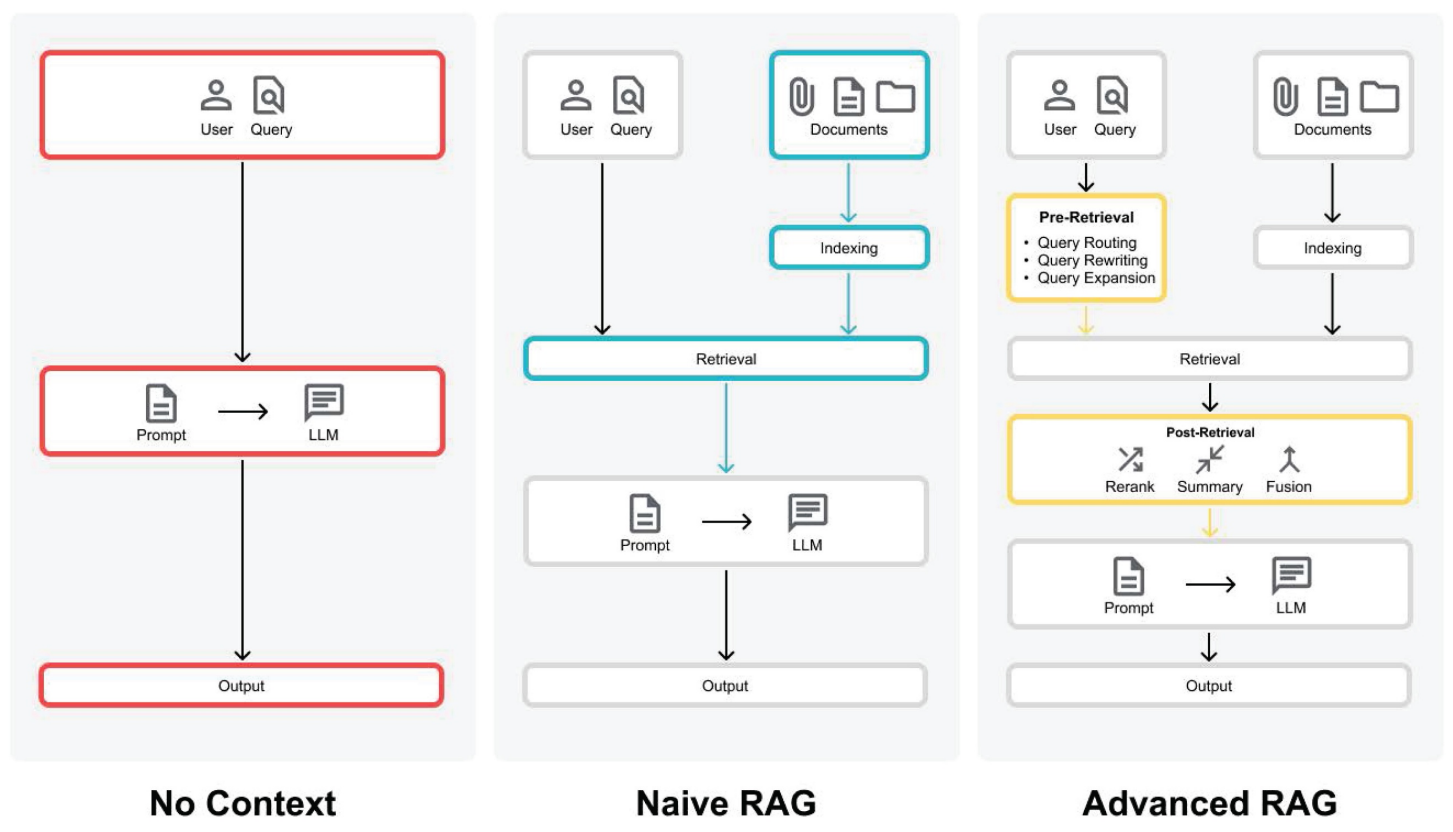
Comparison between LLM and RAGs. (Left) LLM creates the output according to the prompt entered by the user. (Middle) Naive RAG creates the output with additional information from documents. (Right) Advanced RAG creates an output with optimization strategies like pre-retrieval and post-retrieval methods (Figure adapted from Gao et al. (2024)).

In this study, we evaluate two different RAG paradigms: Naive RAGs and Advanced RAGs 9. Naive RAG techniques follow the basic RAG process and only use the research papers without any pre- or post-retrieval methods (see **Figure 2**). However, they face challenges with retrieving the most relevant documents and generating a good answer from them. Advanced RAG techniques use pre- or post-retrieval methods to overcome the challenges of Naive RAGs (see **Figure 2**).

Eibich et al. (2024) investigate the effects of different Advanced RAG techniques on the metrics retrieval precision and answer similarity. Building on this research, we focus on two Advanced techniques: Hypothetical Document Embedding (HyDE) and LLM Reranking (Eibich et al., 2024). HyDE involves creating hypothetical documents that could address a given query and embedding these documents into a vector database. The model then retrieves actual documents that closely match these hypothetical documents in the vector database 8. LLM Reranking involves reordering an initial set of retrieved documents or chunks based on additional queries posed to an LLM 10. This reranking process refines the selection, enhancing the relevance and accuracy of the final output generated by the system.

### LLM Selection and Configuration

For this study, we selected five LLMs to evaluate against each other: OpenAI's GPT-4, and GPT-4O, Cohere's COMMAND R+, Google's GEMINI 1.5, and Antrophic's CLAUDE 3 OPUS. According to the Chatbot Arena by Chiang et al. (2024) these models are among the top 15 available LLMs as of May 2024. The LLMs from OpenAI, Google, and Antrophic were chosen because they rank in the top 5 according to Chiang et al. (2024). Cohere's COMMAND R+ is designed specifically for RAG pipelines, which is why this model was chosen despite not being in the top 10 models.

The field of LLMs is evolving rapidly, with significant improvements occurring over months or even weeks. For instance, the model versions used in our May 2024 evaluation differ from their newer counterparts in terms of capabilities and performance. This rapid evolution presents a methodological challenge for longitudinal comparisons, as newer versions may demonstrate noticeably different performance characteristics.

Nevertheless, our evaluation framework was designed with flexibility and adaptability in mind. It can accommodate new models, different question sets, and varied context documents (see **Figure 1**), making it valuable for ongoing research despite the dynamic nature of LLM development. While direct performance comparisons between different time periods may not be meaningful due to rapid model evolution, the framework itself remains useful for assessing the relative performance of contemporary models and the impact of RAG techniques at any given point in time.

### Evaluation Dataset

We are using a set of 35 questions 12 specifically related to theranostics and nuclear medicine with a particular focus on Peptide Receptor Radionuclide Therapy (PRRT). To evaluate the multilingual capabilities of the LLMs, the questions were translated into both English and German, resulting in a total of 70 questions. These questions address key topics such as therapy, side effects, and costs associated with PRRT for neuroendocrine tumors. While this dataset provides comprehensive coverage of PRRT-related aspects, it has a limited scope regarding other significant theranostic approaches, including radioiodine treatment, PSMA radioligand therapy, mIBG, and FAP. This focused approach was deliberately chosen to enable a deep evaluation within a specific domain, highlighting opportunities for future research to broaden the assessment scope and further validate the framework.

### Response Generation Methods

To evaluate the effect of contextual augmentation we created answers with No Context and answers With Context (see **Figure 2**). For the No Context answers, the LLMs answer the set of evaluation questions based on their pre-trained knowledge. We used the recommended settings from the model providers. To investigate the effects of contextual augmentation through RAG techniques, we use two different RAG paradigms: Naive RAGs and Advanced RAGs (see **Figure 2**).

### Evaluation Methods

**Figure 3:**
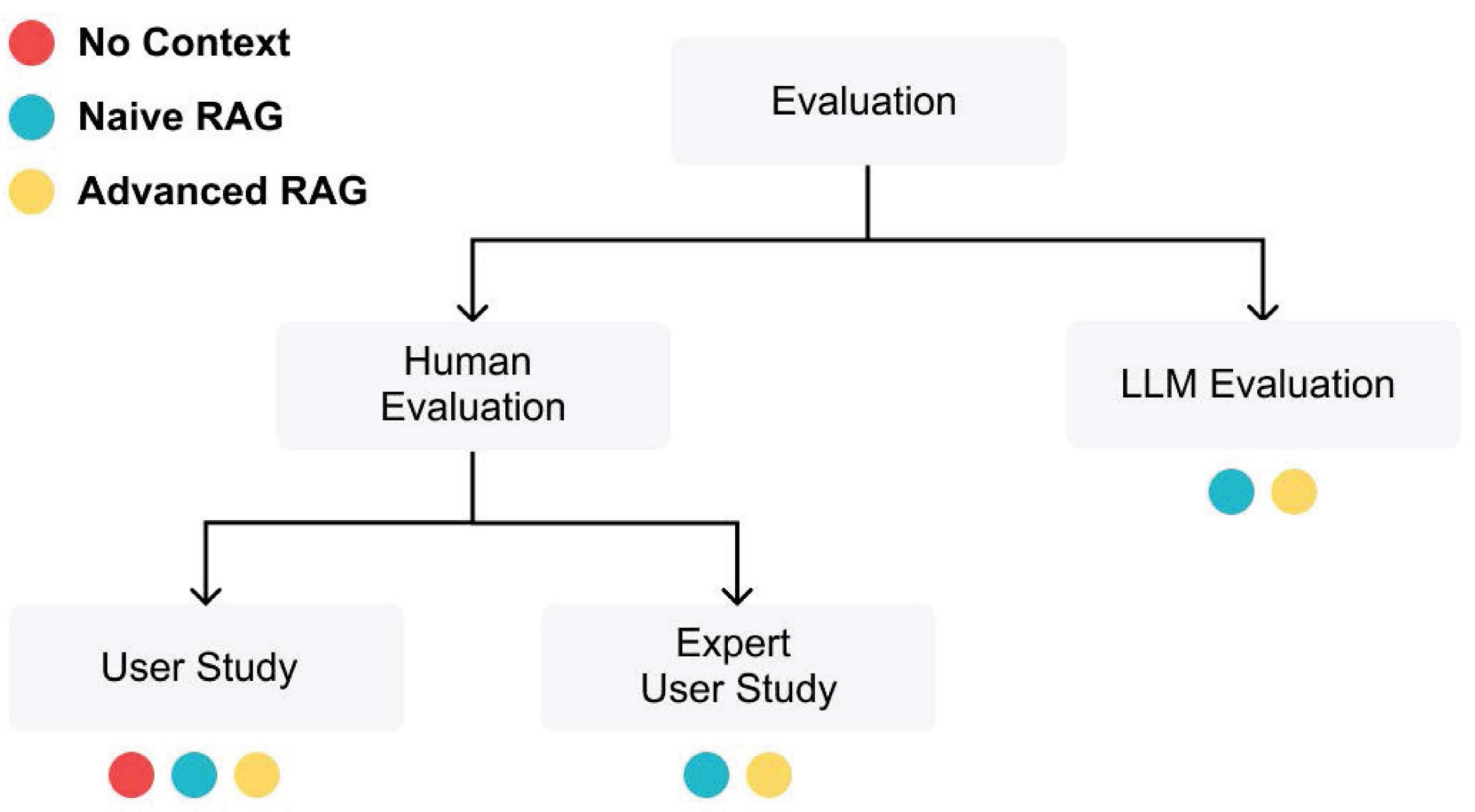
Evaluation process for assessing three LLM answer types: No Context, Naïve RAG, and Advanced RAG. The LLM Evaluation focuses on the effectiveness of contextual augmentation for Naïve and Advanced RAG, while the Human Evaluation includes a general user study for all answer types and an expert study for Naïve and Advanced RAG only.

In our study, we use an approach similar to the one used by Eibich et al. (2024) with the Tonic Validate framework 18 to employ an LLM as an evaluator. Additionally, we conduct human evaluation through a user study, similar to Chiang et al. (2024). LLM evaluations offer scalability, enabling us to perform large-scale comparisons across different models and configurations. Meanwhile, human evaluations provide deep insights into the practical applicability and reliability of the LLMs in real-world medical contexts. Both evaluation methods are designed in a way that makes it easy to replace the evaluated LLMs, which makes it straightforward to test and compare new models as they become available, ensuring the framework remains adaptable and up-to-date with advancements in the field. **Figure 3** illustrates the different evaluation methods and their evaluated answer types.

The primary evaluation method employed in this study is the LLM Evaluator 13. We assessed several key metrics to evaluate the performance of the LLMs: answer consistency, augmentation accuracy, and retrieval precision (see **Table 1**). These metrics specifically focus on evaluating the effectiveness of RAG answers, as they examine how well the LLMs utilize the provided context to enhance their responses. Along with these context-specific metrics, we selected the metrics: language match, language similarity, and word count (see **Table 1**). This evaluation method only assessed the RAG-enhanced answers.

To evaluate all three answer types (No Context, Naïve RAG, Advanced RAG), we conducted two types of human evaluations: a general user study and an expert assessment. Following the methodology of Chiang et al. (2024), participants in both studies compared randomly selected pairs of answers to determine their preferences. This comparative approach, while acknowledging the inherent subjectivity in evaluating responses that may both be technically correct, allowed us to identify patterns in user preferences across different model configurations. The evaluation focused particularly on aspects such as clarity, comprehensiveness, and perceived reliability of the responses, recognizing that preferences might vary based on the evaluator's expertise and specific needs. The primary objective was to rank the No Context answers and then observe any improvements provided by the Naive and Advanced RAG techniques.

However, the direct comparison of No Context answers presented a methodological challenge, as each LLM relies on different pre-trained knowledge bases, making uniform evaluation difficult. Due to a lack of sufficient participants and the large number of generated answers, not all responses could be evaluated by the participants. The user study was conducted with 45 participants. The participants are from various backgrounds, most of them are commercially employed and 6 are healthcare professionals.

Additionally, we conducted a second expert user study to focus solely on selected RAG-generated answers to rank the performance of LLMs using RAG techniques and their ability to extract the relevant information from the provided context. In this survey, we asked experienced HCPs who are experts in theranostics and nuclear medicine, to participate. These experts have between 6 and almost 30 years of experience in this field.

Participants in both user studies were blinded to the model versions they were evaluating. Each participant was presented with responses in a randomized order, ensuring unbiased assessment.

For the evaluation of both user studies, we employed an Elo scoring system, commonly used in ranking games and increasingly used to compare two LLM answers 4,11. The Elo score is recalculated every time the LLM variation "won" or "lost". The score increased or decreased depending on which "players" participated in the "game" between the two models and what their beginning Elo score was. The initial Elo score for all models was 1500.

## Results

### LLM Evaluation Results

**Figure 4:**
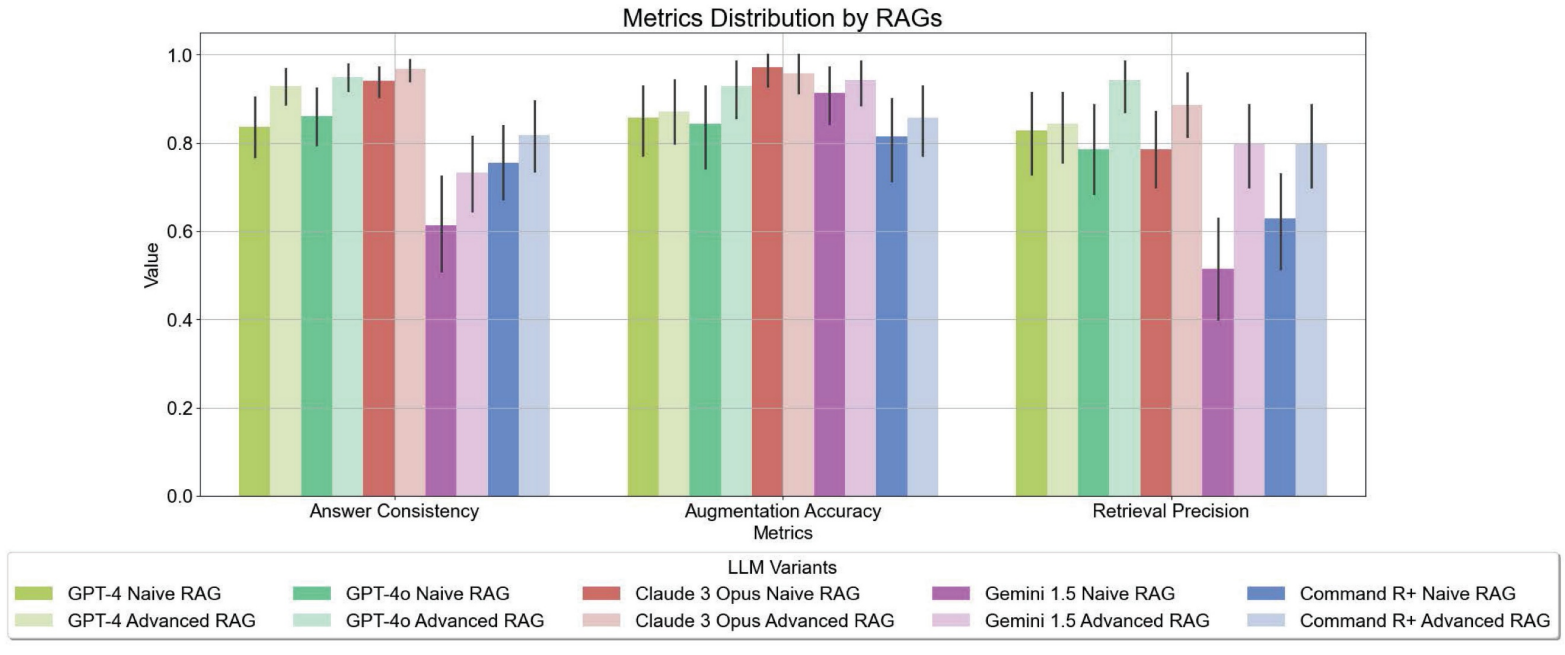
Bar Chart of Mean LLM Response Metrics of the LLM Evaluation - Answer Consistency, Augmentation Accuracy, and Retrieval Precision. These metrics assess the effectiveness of model variants in utilizing additional context to generate responses. The values range from 0 to 1, in indicating the percentage of successfully integrated context in the responses. The higher the value, the better the model is able to use the provided context.

Advanced RAG techniques generally yielded superior results compared to Naive RAG methods. Notably, GEMINI 1.5 and GPT-4O demonstrated the most significant gains in performance with Advanced contextual augmentation techniques (see **Figure 7**). **Figure 4** depicts the distribution of the metrics evaluated with Tonic Validate across various LLM variants utilizing different RAG approaches. Among the models, the best-performing variants in terms of answer consistency and augmentation accuracy are CLAUDE 3 OPUS and GPT-4O. These models consistently achieve the highest scores, indicating robust integration and utilization of contextual information. The most notable improvements between Naive RAG and Advanced RAG are observed in the GEMINI 1.5 and COMMAND R+ variants.

**Figure 5:**
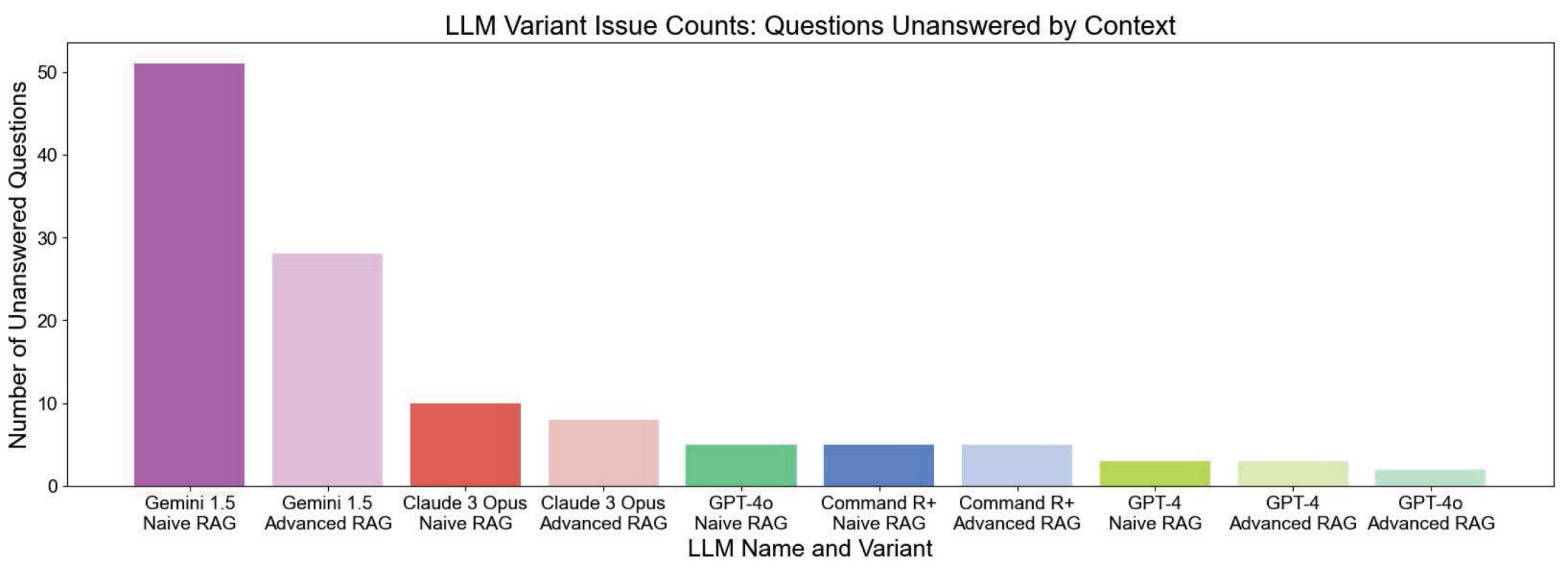
LLM Evaluation - Comparison of LLM Variants in Terms of Issues Related to Unanswered Questions by Context. The bar chart displays the number of unanswered questions out of a total of 70 questions. Higher bars indicate a lower ability to extract relevant information from the context to answer the questions.

LLMs without additional context often "hallucinate" and provide incorrect answers when they do not know the answer, rather than admitting their lack of knowledge. However, LLMs using RAG techniques typically phrase their responses with disclaimers such as, "The context does not provide..." or "The context does not mention...". We filtered the LLM answers by unanswered questions, where the LLM variant could not answer the question even with the provided context (see **Table 2**). **Table 2** serves as a representative case of an unanswered question, illustrating how models indicate when they lack sufficient contextual information. **Figure 5** illustrates the number of unanswered questions. The chart clearly shows that GEMINI 1.5 NAIVE RAG has the highest number of unanswered questions, with 51 unanswered questions out of 70. In contrast, GEMINI 1.5 ADVANCED RAG considerably reduces the number of unanswered questions to around 28. The improvement from Naive RAG to Advanced RAG is not as significant for other LLMs; however, there is always an advantage when using Advanced contextual augmentation. GPT-4O ADVANCED RAG is able to answer all the questions except two.

**Figure 6:**
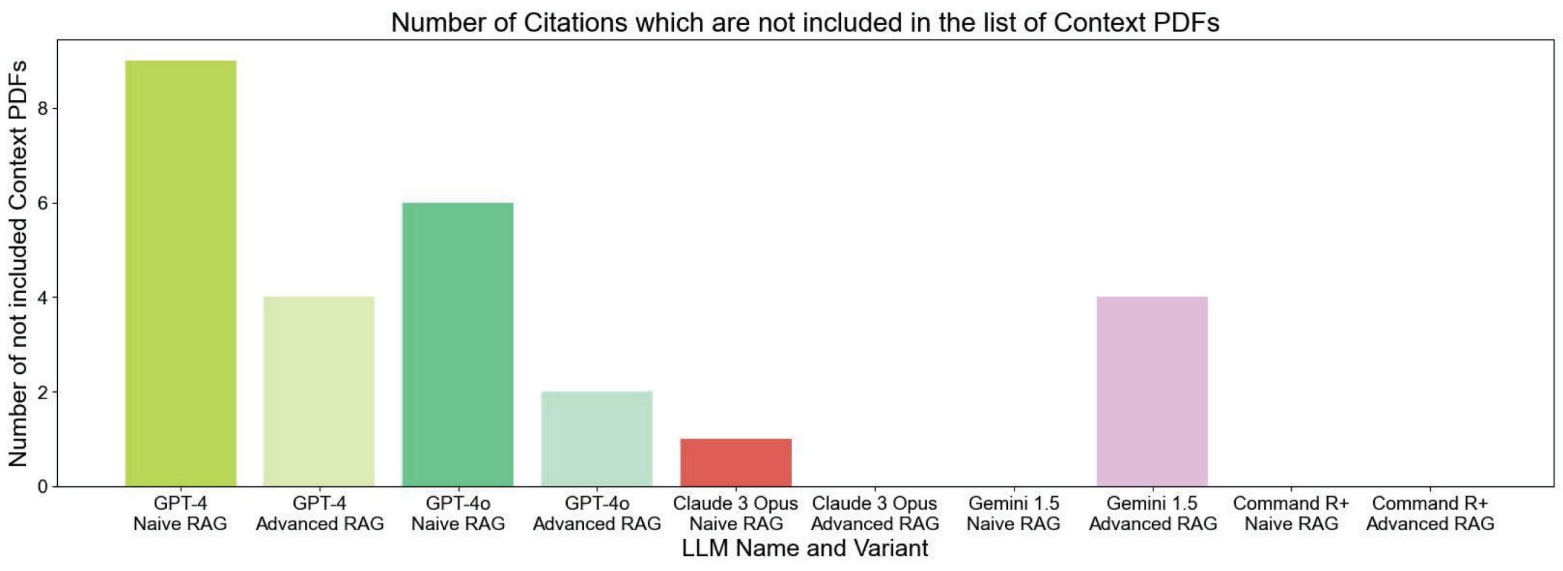
LLM Evaluation - Comparison of LLM Variants in Terms of Issues with Citing Sources Not Included in the Provided Context. The bar chart displays the number of incorrect citations, with higher bars indicating more citations that were not part of the provided context.

Due to the issue of hallucinations in LLMs, we instructed the model in the system prompt to answer in a specific manner, including citing all sources used. We noticed that in some cases, the LLM cites sources, that are not included in the provided list of PDFs, as shown in **Figure 6**. The chart shows that GPT-4 NAIVE RAG has over nine instances where the model cited sources which are not included in the given context. With the Advanced RAG technique, the number reduces to 4. There is an improvement for all the models when using Advanced RAG techniques.

**Figure 7:**
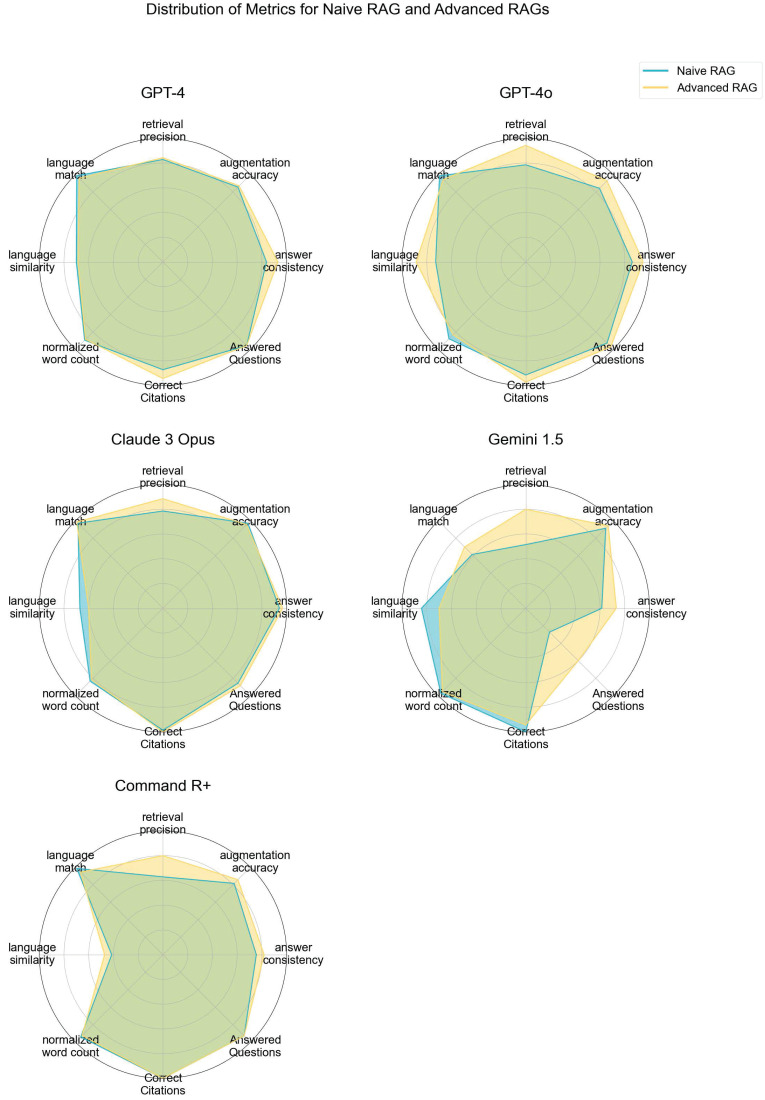
LLM Evaluation - Performance of RAG LLM Variants Across Different Metrics, based on LLM Evaluation. Higher values indicate better performance with contextual augmentation, demonstrating the effectiveness of advanced retrieval and augmentation techniques.

**Figure 7** shows a comparison of the evaluated metrics in a radar chart. GPT-4O and CLAUDE 3 OPUS demonstrate overall good performance in all metrics, whereas GEMINI 1.5 struggles, especially with being able to answer the questions.

### Human Evaluation Results

**Figure 8:**
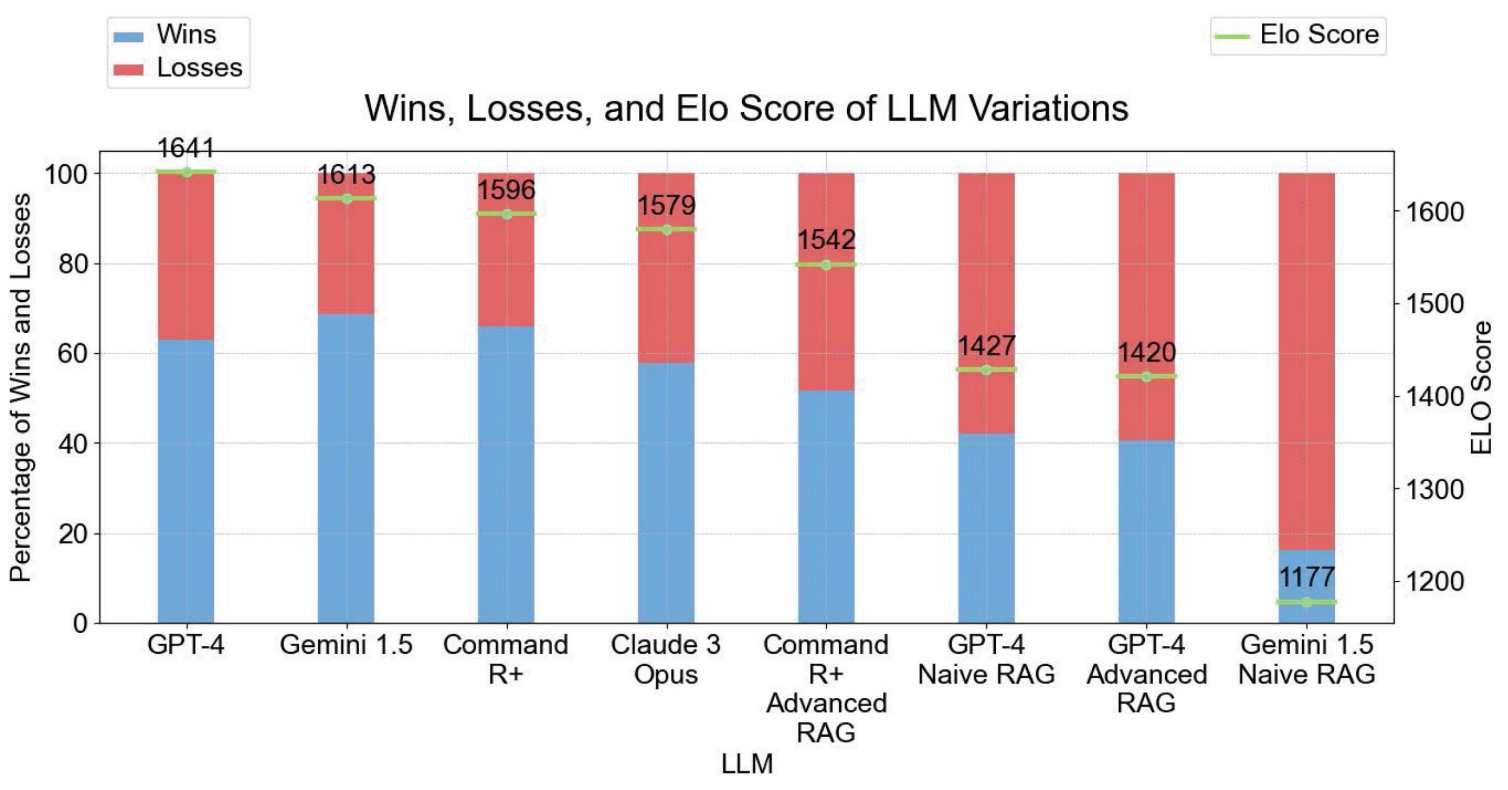
Results of User Study - Wins, Losses, and Elo Scores of LLM Variations. The left y-axis shows the percentage of Wins and Losses. The right y-axis displays the Elo Score with 1641 as the highest Elo Score from GPT-4. All models started with an initial Elo Score of 1500.

**Figure 8** depicts the performance of different LLM variants in terms of their win/loss ratios and Elo scores for the general user study. GPT-4 and GEMINI 1.5 show the highest Elo score with GEMINI 1.5 as the model that wins almost 70% of the "games". Conversely, GEMINI 1.5 NAIVE RAG has the lowest Elo score of 1177, with a win-to-votes ratio of only 16%, highlighting significant difficulties in effectively utilizing context. Other models like Command R+, Claude 3 Opus, and GPT-4O ADVANCED RAG showed varying degrees of performance, with Elo scores ranging from 1542 to 1596. Interestingly the Naive RAG and Advanced RAG variants perform worse than the No Context answer variants in this evaluation.

**Figure 9:**
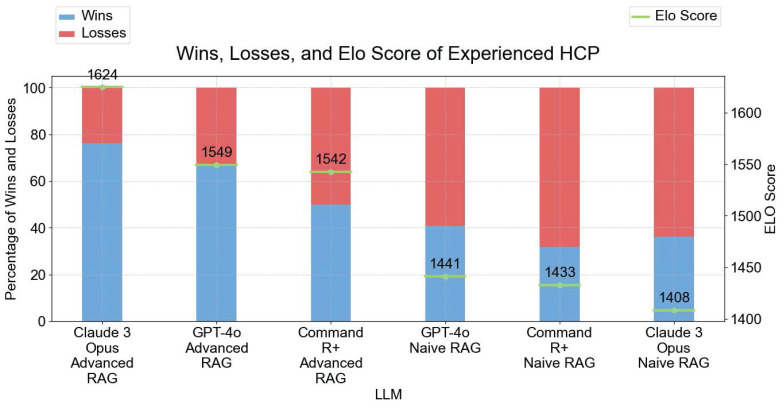
Results of Expert User Study - Wins, Losses, and Elo Scores of LLM Variations. The left y-axis shows the percentage of Wins and Losses. The right y-axis displays the Elo Score with 1624 as the highest Elo Score from Claude 3 Opus ADVANCED RAG. The results from this survey align with the results from the LLM evaluation. All models started with an initial Elo Score of 1500.

In the expert user study we left out the answers from GEMINI 1.5 due to bad performances of the With Context answer types (see **Figure 5**) and the answers from GPT-4 due to the similarity to GPT-4O. There is a clear difference in Elo scores between the Naive RAG answers and the Advanced RAG answers with Advanced RAG variants performing better than the Naive RAG variants (see **Figure 9**).

An additional comparison was made between GPT-4O ADVANCED RAG and COMMAND R+ NAIVE RAG (see **Table 4** and **Table 5**) to a controversial question. The specific comparison was conducted to highlight the distinct advantages of Advanced RAG techniques over Naive RAG in handling complex, domain-specific medical questions. This comparison was chosen because COMMAND R+ is specifically designed for RAG pipelines, making it a suitable baseline for evaluating the impact of more advanced contextual augmentation techniques. Additionally, GPT-4O represents one of the top-performing models in the study, and this head-to-head evaluation helps emphasize how Advanced RAG techniques enhance the quality and comprehensiveness of responses compared to simpler RAG implementations. While the selection may appear arbitrary, it underscores the practical value of refining RAG methods for high-stakes medical applications. In the detailed comparison, 75% of expert participants (3 out of 4) preferred the answers from GPT-4O ADVANCED RAG. However, 2 participants suggested a preference for a combination of answers from both models.

The divergent results between the general user study and expert evaluation require careful analysis. While general users showed a preference for No Context answers and models like GEMINI 1.5, experts and LLM evaluations favored Advanced RAG approaches. This discrepancy may be attributed to several factors, such as differences in evaluation criteria, the impact of domain expertise, or the complexity of answers. General users might prioritize readability and conciseness, while experts focus on technical accuracy and clinical relevance. Experts are better equipped to identify subtle inaccuracies or critical missing information that might not be apparent to general users.

It is worth mentioning that in the general user study, the differences in Elo scores across models are relatively small. 62% of the models have an Elo score above 1500 (Five out of eight models), with a range of just 100 points between the top five models (1641 to 1542, see **Figure 8**). This suggests that general users perceived the models to perform similarly, with readability and accessibility likely influencing their preferences. In contrast, the expert user study results show a more pronounced differentiation. Only 50% of the models achieved an Elo score above 1500 (three out of six models). This highlights the experts' ability to discern more subtle distinctions in performance, particularly regarding technical details and clinical relevance.

The study design intentionally included both general and expert evaluations to capture these differing perspectives, underscoring the importance of balancing technical accuracy with user accessibility when developing medical chatbots.

## Discussion

Theranostic applications often involve complex interactions between imaging modalities, radiopharmaceuticals, and targeted therapies. This field relies on precise and contextually relevant information to optimize patient-specific therapeutic strategies. Adding relevant contextual information, particularly through Advanced RAG techniques, substantially improves key metrics such as answer consistency, augmentation accuracy, and retrieval precision. This demonstrates that Advanced contextual augmentation methods not only enhance the accuracy of the provided information but also ensure that responses are more contextually relevant and reliable. Given the dynamic and rapidly evolving nature of theranostics, ensuring that chatbot responses are contextually relevant and accurate is essential for effective patient management and decision-making. Therefore, it is important that the model used for a theranostic chatbot can deliver accurate answers, utilize relevant sources, and address most of the questions posed.

The study's findings underscore the critical importance of employing Advanced retrieval and augmentation techniques to enhance LLM performance. For instance, GEMINI 1.5 NAIVE RAG showed lower performance across all metrics, particularly in augmentation accuracy and retrieval precision. However, with the Advanced RAG approach, there was a substantial improvement in these metrics, demonstrating that advanced retrieval and augmentation techniques enhance the model's ability to generate accurate and contextually relevant responses. As illustrated in **Table 3** compared to **Table 2**, GEMINI ADVANCED RAG is notably more effective in answering questions with the provided context. Overall, the data highlights that all models benefit from Advanced RAG techniques. Specifically, Advanced RAG techniques enhance LLM performance by improving the accuracy and relevance of responses, reducing errors, increasing the number of answered questions, and optimizing the use of retrieved context. The radar charts (see **Figure 7**) demonstrate that models like GPT-4O and CLAUDE 3 OPUS exhibit strong performance across all metrics, nearly filling the entire circle, indicating a well-rounded capability in handling complex queries. In an additional comparison between GPT-4O and COMMAND R+ on a controversial question, experts suggested a combination of both models (see **Table 4** and **Table 5**). This indicates that human subjectivity should be considered in evaluations, as each model has unique strengths that could be leveraged together for optimal performance.

Despite promising results, there are several limitations. LLM-based evaluations are limited by the quality and biases of the models themselves and the data they are trained on. A significant challenge lies in comparing human and LLM evaluations, particularly due to the difficulty of uniformly assessing the No Context answers. The Tonic Validate framework focuses exclusively on With Context answers, complicating direct comparisons. To address this, future studies could adjust the number of evaluated models or involve a larger participant pool in the user study to improve robustness. Another limitation is the potential for missing information within the 197 context papers, which may influence LLM performance. A key limitation of this study is its primary focus on PRRT-related questions, which represents only one aspect of the broader theranostics field. While this focused approach allowed for detailed evaluation within a specific domain, it leaves room for expansion into other crucial areas of theranostics.

This study serves as an important proof of concept for theranostic chatbots, demonstrating that contextual augmentation through Advanced RAG techniques can significantly improve the accuracy and relevance of LLM responses in this specialized domain. While the selected research papers focused primarily on PRRT-related topics, this focused approach allowed for a thorough initial validation of the methodology. Notably, doubling the number of questions would likely not fundamentally alter the validation results, as the current dataset already provides sufficient evidence of the framework's effectiveness. The rapid evolution of LLM capabilities - with models showing significant improvements over months or even weeks - presents both an opportunity and a methodological challenge. This dynamic nature of the field means that direct performance comparisons across different time periods may not be meaningful. However, the framework's design, with its interchangeable components for questions, models, and context documents, ensures its continued relevance for evaluating contemporary models and RAG techniques.

A comprehensive follow-up study could include questions covering additional theranostic approaches such as radioiodine treatment, or PSMA radioligand therapy, potentially incorporating clinical case scenarios and imaging-specific queries and comparing the evolution of the model's performances over time. Such expansion would provide a more complete understanding of LLM capabilities across the full spectrum of theranostic applications. Addressing these limitations and exploring new techniques will further enhance LLM performance and their application in various domains.

## Conclusion

This study marks a significant milestone as a proof of concept for theranostic chatbots, demonstrating the potential of contextual augmentation through Advanced RAG techniques to enhance medical chatbot performance. While the research focused primarily on PRRT-related queries, this deliberate scope limitation allowed for thorough validation of the methodology. The results convincingly show that Advanced techniques notably outperform traditional methods, particularly when applied to models like GPT-4O and CLAUDE 3 OPUS. Importantly, the framework's modular design - allowing for interchangeable questions, models, and context documents - ensures its adaptability despite the rapidly evolving nature of LLM capabilities. This adaptability is crucial given the frequent updates and improvements in LLM technology, where model capabilities can change significantly within months.

Despite these advancements, the theranostic chatbot is not yet perfect. The next steps should be to ensure that medical chatbots provide accurate, reliable, and contextually relevant information. Future work should expand the evaluation scope beyond PRRT to encompass the full breadth of theranostic applications, while carefully considering the methodological challenges posed by rapidly evolving LLM capabilities. This expansion would provide a more comprehensive assessment of how contextual augmentation techniques perform across different theranostic domains and clinical scenarios. Additionally, addressing biases, increasing transparency in AI decision-making processes, integrating user feedback for continuous improvement, and ensuring compliance with regulatory standards are crucial. These measures will help build trust, enhance user acceptance, and ensure that theranostic chatbots provide accurate, reliable, and contextually relevant information.

## Supplementary Material

Supplementary appendix: question list.

## Figures and Tables

**Table 1:** Metrics used in LLM-based evaluation to assess the quality of model responses

Answer Type	Metric	Range	Measurement
Naive RAG, Advanced RAG	Answer consistency	0 - 1	The percentage of the LLM answer that can be attributed to retrieved context.
Naive RAG, Advanced RAG	Augmentation accuracy	0 - 1	The percentage of retrieved context which appears in the LLM answer.
Naive RAG, Advanced RAG	Retrieval precision	0 - 1	Is the retrieved context relevant to answer the given question?
No Context, Naive RAG, Advanced RAG	Language match	0 - 1	Is the LLM answer in the expected language?
No Context, Naive RAG, Advanced RAG	Language similarity	0 - 1	Are the German and English answers similar?
No Context, Naive RAG, Advanced RAG	Word count	0 - 485	Number of words in the LLM answer

**Table 2:** Naive RAG Answer from Gemini 1.5 Naive RAG. Example of an unanswered question response from GEMINI 1.5 Naive RAG, illustrating how models indicate when they cannot provide information from the given context.

What equipment does a clinic need to perform PRRT?
This context does not mention anything about the equipment needed to perform PRRT, so I cannot answer this question from the provided context.

**Table 3:** Advanced RAG Answers from GEMINI 1.5: Example of the same question from Table 2, demonstrating how the model, using Advanced RAG, provides a suitable and accurate response.

What equipment does a clinic need to perform PRRT?
To perform PRRT, a clinic needs the following equipment: **Prophylactic Protective Covering**: For patient stretchers, chairs, floors, and lower walls to reduce potential contamination from radioactive body fluids (Hope et al., 2019, p. 2). **Treatment Suite**: Ideally equipped with necessary facilities to handle radioactive materials and patient care (Hope et al., 2019, p. 2). **Radiation Safety Equipment**: To ensure proper precautions during the administration of radioactivity (Hope et al., 2019, p. 2). **Infusion Equipment**: For administering amino acids and antiemetics, as well as the radiopharmaceutical 177Lu-DOTATATE (Hope et al., 2019, p. 2). **Monitoring Equipment**: For patient monitoring during and after the treatment (Hope et al., 2019, p. 2). These elements are essential to ensure the safe and effective administration of PRRT.

**Table 4:** Answer generated by GPT-4O ADVANCED RAG to a controversial medical question, showcasing the benefits of advanced retrieval techniques in delivering detailed, accurate, and well-structured responses.

Is it possible to compare the different guidelines for the management of neuroendocrine tumors (ENETS, NANETS)? Where are the differences in the therapy of this disease?
Yes, it is possible to compare the different guidelines for the management of neuroendocrine tumors (NETs) between the European Neuroendocrine Tumor Society (ENETS) and the North American Neuroendocrine Tumor Society (NANETS). While both organizations provide guidelines for the management of NETs, they may differ in their specific recommendations and areas of focus due to varying expert opinions and regional considerations. Some key differences between ENETS and NANETS guidelines include: ENETS guidelines tend to be more comprehensive and detailed, covering various aspects of NET management, including specific recommendations for different types of NETs (such as gastroenteropancreatic and bronchial NETs) and even providing guidelines for managing metastases to specific organs, like the peritoneum, bone, and lung. NANETS guidelines, on the other hand, often focus on more generalized surveillance and medical management strategies for NETs, particularly for midgut neuroendocrine tumors. ENETS guidelines are primarily European-focused, while NANETS guidelines are geared towards a North American context, taking into account regional differences in healthcare systems, available treatments, and patient populations. The timing of updates and the incorporation of new research findings may differ between the two organizations, leading to variations in their recommendations over time. It is important for clinicians to be aware of these differences and to refer to the most up-to-date guidelines from both organizations when managing patients with NETs, adapting their approach based on individual patient needs and the specific context of their healthcare setting. References: Pavel, M., Falconi, M., Eriksson, B., Kaltsas, G., Rindi, G., Ruszniewski, P., ... & Capdevila, J. (2016). ENETS Consensus Guidelines for the management of patients with digestive neuroendocrine neoplasms: well-differentiated pancreatic non-functioning/functioning tumors and type 1 and type 2 gastric NETs; duodenal, small intestinal, colorectal and unknown primary tumors. Neuroendocrinology, 103(2), 172-185. Strosberg, J. R., Halfdanarson, T. R., Bellizzi, A. M., et al. The North American neuroendocrine tumor society consensus guidelines for the management of neuroendocrine tumors. Pancreas, 46(6), 707-714, 2017.

**Table 5:** Answer generated by COMMAND R+ NAIVE RAG to the same question, highlighting the limitations of basic retrieval methods in providing depth and accuracy compared to advanced approaches.

Is it possible to compare the different guidelines for the management of neuroendocrine tumors (ENETS, NANETS)? Where are the differences in the therapy of this disease?
Yes, it is possible to compare the different guidelines for the management of neuroendocrine tumors (NETs) provided by the European Neuroendocrine Tumor Society (ENETS) and the North American Neuroendocrine Tumor Society (NANETS). Both sets of guidelines offer comprehensive recommendations for the diagnosis, treatment, and follow-up of NETs, but there are some differences in their approaches and specific recommendations. ENETS guidelines emphasize the importance of a multidisciplinary tumor board for therapeutic decision-making, especially to exploit surgical therapy (Pavel et al., 2016). They provide detailed algorithms for the management of liver metastases, including options like surgery, radiofrequency ablation (RFA), transarterial chemoembolization (TACE), and selective internal radiation therapy (SIRT), which is still considered investigational (Pavel et al., 2016). ENETS also highlights the use of somatostatin analogues (SSA), sunitinib, and everolimus, which have been evaluated in placebo-controlled trials and approved for antiproliferative therapy in different NET subtypes (Pavel et al., 2016). NANETS guidelines, while similar in many respects, may have different recommendations based on regional practices and available treatments. For instance, the use of specific drugs or therapeutic approaches might vary due to differences in drug approval status or clinical trial availability in North America compared to Europe. In summary, while both ENETS and NANETS provide robust frameworks for managing NETs, differences may arise in specific therapeutic recommendations, the emphasis on certain treatment modalities, and the integration of new evidence from clinical trials.

## Abbreviations

AI: Artificial Intelligence
RAG: Retrieval Augmented Generation
LLM: Large Language Model
HCPs: Health Care Professionals
