# Supplementary material for: Optimizing theranostics chatbots with context-augmented large language models
Source: Theranostics. 2025 Apr 21;15(12):5693–704. doi: 10.7150/thno.107757 (PMC12068303; doi:10.7150/thno.107757)
Supplement: Supplementary file 1 — Supplementary appendix: question list. [file thnov15p5693s1.pdf]

APPENDIX

Questionlist

1. What is the role of PRRT in GEP-NET?
2. For which indication is PRRT approved?
3. What is the place of using PRRT in the surgical setting in GEP-NET?
4. What is Peptide Receptor Radionuclide Therapy (PRRT)?
5. How many PRRT treatments can you have?
6. How much does a PRRT treatment cost?
7. Which isotopes are used for therapy and diagnostics in PRRT?
8. Is PRRT harmful for the patient?
9. Is PRRT the future for metastatic cancer patients?
10. What equipment does a clinic need to perform PRRT?
11. Which doctors perform PRRT? Do you have to be a nuclear medicine physician?
12. What are the most common side effects of PRRT?
13. What are the benefits of PRRT?
14. What are the challenges of PRRT?
15. What are the most important inclusion/exclusion criteria of the COMPETE trial?
16. What are the most important inclusion/exclusion criteria of the COMPOSE trial?
17. Which radiopharmaceutical drugs are approved by the FDA?
18. In which indications are PSMA-targeting radiopharmaceuticals currently being developed?
19. What are the major studies that have been initiated or conducted in the field of neuroendocrine tumors in recent years?
20. What is the control arm in the Netter-1 trial?
21. What is the control arm in the Netter-2 trial?
22. What is the control arm in the COMPETE trial?
23. What is the control arm in the COMPOSE trial?
24. Is it possible to compare the different guidelines for the management of neuroendocrine tumors (ENETS, NANETS)? Where are the differences in the therapy of this disease?
25. In which section are PRRT therapies presented in the ESMO Guidelines?
26. What types of neuroendocrine tumors are investigated in the COMPETE trial?
27. What was the median PFS in the Netter-2 trial?
28. In which indications does ITM Radiopharma research radiopharmaceuticals?
29. Which isotopes are used in ITM Radiopharma's studies?
30. When was ITM Radiopharma founded?
31. Which products does ITM Radiopharma already have on the market?
32. Which congresses does ITM Radiopharma participate in?
33. How does PRRT work/what is the drug made up of?
34. What is the major requirement for a patient to be treated with PRRT?
35. What is theranostics?
